# *Distantiae* Transmission of *Trypanosoma cruzi*: A New Epidemiological Feature of Acute Chagas Disease in Brazil

**DOI:** 10.1371/journal.pntd.0002878

**Published:** 2014-05-22

**Authors:** Samanta Cristina das Chagas Xavier, André Luiz Rodrigues Roque, Daniele Bilac, Vitor Antônio Louzada de Araújo, Sócrates Fraga da Costa Neto, Elias Seixas Lorosa, Luiz Felipe Coutinho Ferreira da Silva, Ana Maria Jansen

**Affiliations:** 1 Laboratory of Trypanosomatid Biology, Oswaldo Cruz Institute, FIOCRUZ, Rio de Janeiro, Rio de Janeiro, Brazil; 2 Laboratory of Biology and Parasitology of Wild Reservoir Mammals, Oswaldo Cruz Institute, FIOCRUZ, Rio de Janeiro, Rio de Janeiro, Brazil; 3 International and National Laboratory of Reference for Triatominae Taxonomy, Oswaldo Cruz Institute, FIOCRUZ, Rio de Janeiro, Rio de Janeiro, Brazil; 4 Laboratory of Cartography, Military Institute of Engineering, IME, Rio de Janeiro, Rio de Janeiro, Brazil; René Rachou Research Center, Fiocruz, Brazil

## Abstract

**Background:**

The new epidemiological scenario of orally transmitted Chagas disease that has emerged in Brazil, and mainly in the Amazon region, needs to be addressed with a new and systematic focus. Belém, the capital of Pará state, reports the highest number of acute Chagas disease (ACD) cases associated with the consumption of açaí juice.

**Methodology/Principal Findings:**

The wild and domestic enzootic transmission cycles of *Trypanosoma cruzi* were evaluated in the two locations (Jurunas and Val-de Cães) that report the majority of the autochthonous cases of ACD in Belém city. Moreover, we evaluated the enzootic cycle on the three islands that provide most of the açaí fruit that is consumed in these localities. We employed parasitological and serological tests throughout to evaluate infectivity competence and exposure to *T. cruzi*. In Val-de-Cães, no wild mammal presented positive parasitological tests, and 56% seroprevalence was observed, with low serological titers. Three of 14 triatomines were found to be infected (TcI). This unexpected epidemiological picture does not explain the high number of autochthonous ACD cases. In Jurunas, the cases of ACD could not be autochthonous because of the absence of any enzootic cycle of *T. cruzi*. In contrast, in the 3 island areas from which the açaí fruit originates, 66.7% of wild mammals and two dogs displayed positive hemocultures, and 15.6% of triatomines were found to be infected by *T. cruzi*. Genotyping by mini-exon gene and PCR-RFLP (1f8/Akw21I) targeting revealed that the mammals and triatomines from the islands harbored TcI and *Trypanosoma rangeli* in single and mixed infections.

**Conclusion/Significance:**

These findings show that cases of Chagas disease in the urban area of Belém may be derived from infected triatomines coming together with the açaí fruits from distant islands. We term this new epidemiological feature of Chagas disease as “*Distantiae* transmission”.

## Author Summary

After the interruption of *Trypanosoma cruzi* transmission by *Triatoma infestans*, a new epidemiological scenario emerged in Brazil: the oral route. In the Amazon region, considered as a non-endemic Chagas disease region, *T. cruzi* is thought to exist as an enzootic parasite of wild animals. Currently, this region comprises approximately 92.3% of human Chagas cases in Brazil, the majority of which are in the Pará state. Its capital, Belém, reports the highest number of acute Chagas disease (ACD) cases (associated with the consumption of açaí juice), despite being an urban environment without the domiciliation of triatomines or an enzootic cycle. Thus, we decided to test the hypothesis that the cases related to the urban area of Belém are derived from infected sylvatic triatomine bugs accidentally transported from the islands that provide the majority of the açaí fruit sold in the metropolitan area. We term this new epidemiological feature “*Distantiae* transmission,” indicating that the ACD cases in the metropolitan region of Belém are related to infected triatomines transported from the islands. In the Amazon region, food contamination by infected triatomines is the result of the unhygienic manipulation of açaí and is supposed to be controlled by an epidemiological and sanitary surveillance program.

## Introduction

*Trypanosoma cruzi* (Chagas 1909), the etiologic agent of Chagas disease [1], is a protozoan parasite included in the Trypanosomatidae family, order Trypanosomatida [2]. This successful parasite species is found from the southern United States to Argentinean Patagonia [3]. Trypanosomiasis due to *T. cruzi* is characterized as a complex zoonosis, transmitted by approximately 130 species of triatomines from the Reduviidae family (Hemiptera insects that are exclusively hematophagous at all life stages) and able to infect more than 150 species of wild and domestic mammals in 8 orders [4].

*T. cruzi* is highly adapted to the parasitic way of life, so it is able to colonize nearly all tissues of its hundreds of mammalian host species. Triatomines are generalistic and ubiquitous vectors, so *T. cruzi* transmission cycles that may occur in all forest strata according to the extant mammalian fauna. Additionally, *T. cruzi* displays an impressive heterogeneity, and currently six discrete typing units (DTUs) are recognized, in addition to the newly identified TcBat genotype, so far described as restricted to bats [5]–[7]. These DTUs establish peculiar interactions with their hosts in distinct time-space scales. Recent studies have also reported intraspecific variability within these genotypes, such as in TcI isolates [8]. The cycles of *T. cruzi* transmission can be considered as complex systems due to their non-linearity, unpredictability and multivariable nature.

Classically, human beings were infected by *T. cruzi* after the introduction of infected bug feces into a skin lesion (or mucosa) after a blood meal by domiciliated triatomine bugs. The current emergence, or re-emergence, of Chagas disease cases with a new epidemiological profile (transmission to humans is currently independent of the domiciliation of triatomines in many regions) highlights the importance of the evaluation of this zoonosis by public health authorities from a new epidemiological perspective [9]. American trypanosomiasis is primarily and essentially a sylvatic parasitosis, and this characteristic urgently deserves even greater attention, given the control of domiciliary transmission by *Triatoma infestans*. In this sense, it is of fundamental importance to understand the ecology of the transmission cycles of *T. cruzi*, taking into account environmental variables, the cultural characteristics of local populations, wild and domestic mammalian species' composition and triatomine species to clarify the ecological system responsible for parasite maintenance in areas that face cases/outbreaks of ACD.

*T. cruzi* is thought to have existed as an enzootic pathogen of wild animals in the Amazon Region for millions of years. Since the early 20th century, several studies have been conducted to identify the hosts and reservoirs of this parasite and of the triatomine vectors responsible for its transmission in the Brazilian Amazon. More than one hundred mammalian species in the region have been identified as being naturally infected by *T. cruzi* [10], [11]. *Triatoma infestans* has never been documented in ACD outbreaks, and native vectors are not found in domiciliary colonies. Due to this feature, the Amazon region has been incorrectly interpreted as a non-endemic Chagas disease region. In fact, the first autochthonous human case in the region was recorded only in 1969, in the city of Belém, state of Pará [12], and since then, autochthonous cases have been reported in increasing numbers. However, it was only after 2005, when transmission decreased in other parts of the country (after the recognition of the control of *T. infestans* by the World Health Organization, [13]) and surveillance in the Amazon region was intensified (through the training of malaria technicians for *T. cruzi* identification in blood smears), that microepidemics of ACD began to be reported regularly and frequently. These microepidemics were mainly due to oral transmission and were associated with the consumption of the juice of the palm-tree fruit açaí.

The oral transmission of *T. cruzi* is currently predominant, accounting for 62% of cases in Brazil (93% considering only the Amazon region) between 2006 and 2012 (data from the Information System for Noticeable Diseases - SINAN). It is worth mentioning that cases from the Amazon region occur independently of the domiciliation of triatomines (which was never described in this region), are always the result of exposure to the *T. cruzi* sylvatic cycle and are more frequent in familial groups living near the forest. Another peculiar fact is the seasonality of these orally transmitted ACD cases, as the majority of cases coincide with the harvest of açaí from August to November, when pluvial indexes are lower. This trait suggests that there is a direct relationship between disease incidence and the consumption of the improperly manipulated fruit (data from the Secretary of Health from Pará State).

For certain outbreaks associated with the consumption of açaí juice in Pará and Amapá states, it has already been proposed that infected insects, which frequently nest in the açaí palm tree, might be transported from the açaí fruit collecting area, via panniers and sacks, to the juice extraction machines in other areas, allowing human infection due to ingestion of the *T. cruzi*-contaminated açaí juice [14], [15]. Infected triatomines may be crushed in the juice extraction machines due to a lack of sanitary care during juice preparation, leading to contamination of the juice and to cases of Chagas disease, as proposed by Valente and coauthors [15], [16].

Most of the açaí fruit consumed in Belém arrives at a commercial and historical warehouse (denominated Ver-o-Peso) daily, coming from islands near Belém, where fruit processors buy and carry the fruit to the place where they will prepare and sell the açaí pulp juice. Aiming to test the hypothesis first suggested by Valente and coauthors [16], our group proposed that the cases related to the urban area of Belém are derived from infected triatomines coming from the islands that supply the city with the fruits and that are up to 1.5 km away from the metropolitan area. We termed this new epidemiological feature *Distantiae* Transmission. To test our hypothesis, we evaluated the enzootic transmission cycle of *T. cruzi* in the two locations that report the majority of the autochthonous cases in the metropolitan region of Belém (Val-de-Cães and Jurunas) and on the three islands that provide the majority of the açaí fruit that is sold in the metropolitan area (Combu, Murutucu and Pato). In each locality, we evaluate the *T. cruzi* transmission among triatomines and wild and domestic mammals and the role played by each of them in the transmission of the parasite.

## Methods

### Geographic origin

The Belém municipality, capital of Pará state, is located in northern Brazil (1°27′21″S and 48°30′14″W) and consists of an area of 1,059,402 km^2^. The population is estimated to include 1,381,475 living in the urban area of the municipality and only 11,924 living in the rural area (Brazilian Institute of Geography and Statistics IBGE, census 2010). Belém is known as the “Metropolis of the Amazon region” because it is the second most populous city of the northern region (1,315.26 habitants/km^2^) and represents the second most important economic center of the Brazilian Amazon region.

The climate is hot and humid, or typically equatorial, and is directly influenced by the Amazon rainforest, where rains are constant. The pluviometric index is 2889 mm/year, and the average annual temperature is 26°C, reaching up to 35°C in the warmer months from July to November. The urban portion of the municipality is surrounded by peripheral areas that sporadically display remaining patches of the Amazonian forest.

**Figure 1:**
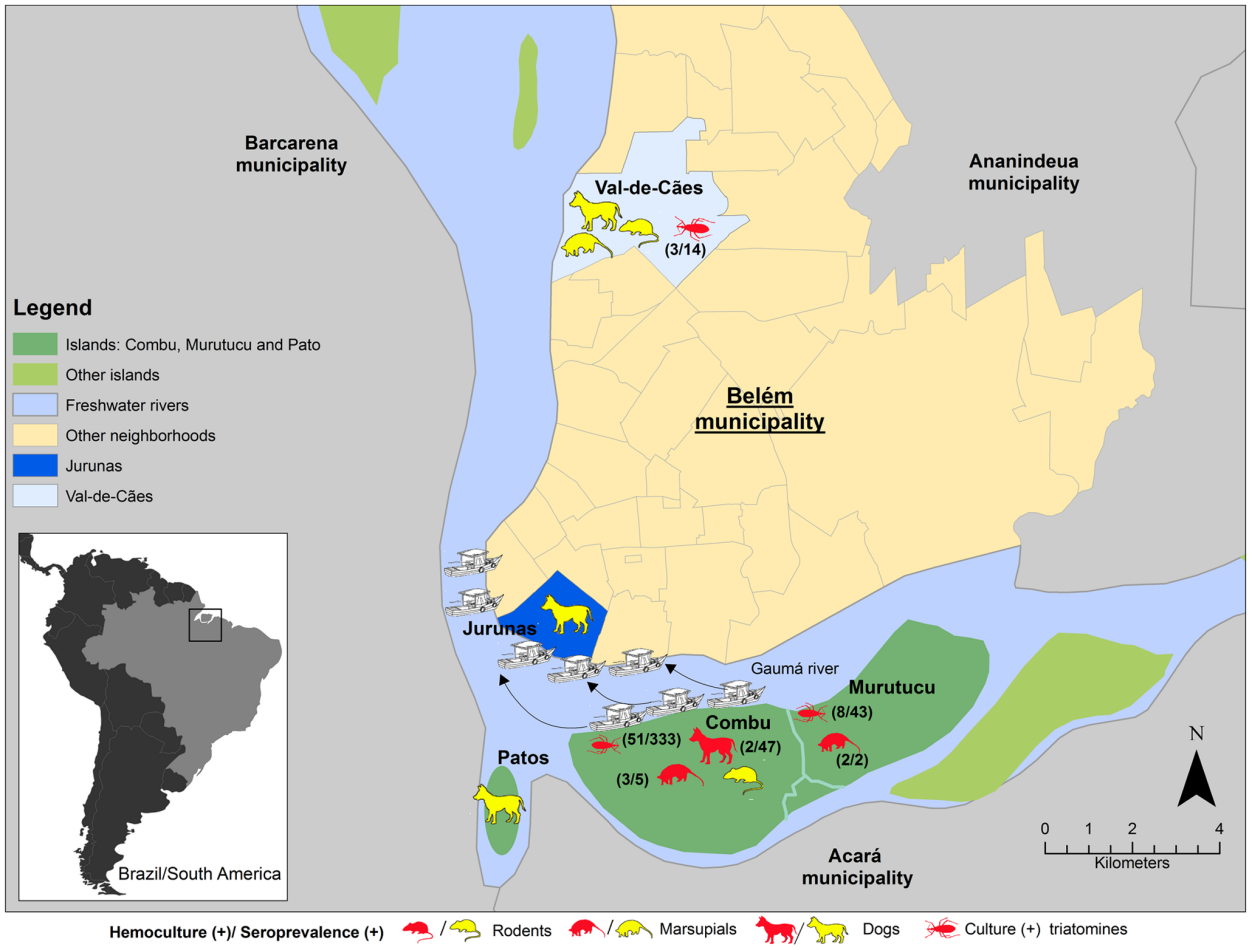
Map of the spatial distribution of the *Trypanosoma cruzi* infections. Mammals and triatomines collected in the Belém municipality, in the urban portion (Jurunas and Val-de-Cães) and on the islands (Combu, Murutucu and Pato). The pictures framed in red represent infections animals (positive cultures) among the triatomines, dogs and small mammals examined, and the pictures framed in yellow represent positive serology. On the left of the figure is the study site in Brazil, highlighting Pará state and the Brazilian and South American borders.

The two evaluated places in the metropolitan region of Belém have distinct levels of urbanization: Jurunas is a heavily urbanized suburb, whereas Val-de-Cães, despite also presenting an intense process of urbanization, still maintains forest areas. The opposite situation is observed in the studied islands, which are scarcely populated, suffering only minor anthropic action, and which still maintain certain characteristics typical of the Amazonian forest. Outside of the metropolitan area, but still within the political limits of the municipality, these islands are composed of freshwater swamp forest between diverse riverbanks and parts of the original and secondary vegetation (Amazonian forest). The three evaluated islands (Combu, Murutucu and Pato) are sparsely occupied, and their vegetation is composed of typical Amazonian forest, with restricted exploration where fruit (mainly açaí) collection occurs in addition to subsistence plantation. These islands are at a mean distance of 1.5 km south of the urban area of Belém and are situated on the left bank of the river Guamá. The ecosystem is under the direct influence of tidal rivers, displaying constant flooding, and is hence characterized as lowland soil (flood plain) (Figure 1). Expeditions were performed between July 2009 (urban section) and November 2011 (islands), all in dry seasons. In all of these places, vectors and mammals (wild, synanthropic and domestic) were investigated for *Trypanosoma cruzi* infection.

According to data from SINAN (Information System for Noticeable Diseases), 1529 cases of ACD were recorded in Brazil between 2006 and 2012, with approximately 71.9% (N = 1100) of them concentrated in the Amazon region. The state of Pará was responsible for more than half of the cases in Brazil (65.9%) and for approximately 92.3% of cases in the northern region of Brazil. The mortality rate between 2010 and 2011 was 6.2%, and in 2012, 3 deaths were recorded. Of the cases reported in Pará state, one fifth occurred in the capital Belém (20.3%; N = 189), 19.4% of which occurred in the neighborhoods Jurunas (14.1%; N = 24) and Val-de-Cães (5.2%; N = 9).

### Sample collection

#### Small wild mammal capture

The capture of small wild and synanthropic mammals was performed as follows. Live traps were arranged in linear transects (all transects initiated in the peridomestic environment of houses), and the capture points were established with intercalated traps. The traps were baited with a mixture of peanut butter, banana, oats and bacon and set at 20-m intervals in all types of vegetation formations and habitats. The total capture efforts were 820 traps-night for the islands (Combu, Murutucu and Pato) and 827 traps-night for the urban section (807 in Val-de-Cães and 20 in Jurunas). In Jurunas, due to a high degree of devastation and a lack of forested areas, it was not possible to establish transects, and the traps were arranged in four residences in which the dwellers reported the presence of synanthropic mammals. For all captured small mammals, morphological characteristics and body measurements were recorded for age estimation and taxonomic identification. The taxonomic status of rodents was subsequently confirmed by karyological analyses [17]. Wild mammals were anesthetized (9∶1, ketamine cloridrate 10% and acepromazine 2%) and had their blood collected by cardiac puncture in a field laboratory set up exclusively for this purpose.

#### Ethics statement

All wild animal manipulation procedures were performed in accordance with the COBEA (Brazilian College of Animal Experimentation) following the guidelines of the Animal Ethics Committee (CEUA) protocol of FIOCRUZ (Oswaldo Cruz Institute Foundation), Ministry of Health, Brazil. All procedures followed protocols approved by the FIOCRUZ Committee of Bioethics (license 0015-07), and the wild animal captures were licensed by the Brazilian Institute of Environment and Renewable Natural Resources (IBAMA/CGFAU/LIC) – permanent license 3665-1.

#### Dog survey

The active search for dogs was conducted in the houses neighboring the linear transects where the small wild and synanthropic mammals were captured. In all cases, with the informed consent of their owners, who also helped us to handle the animals, blood was collected by puncture of the radial or femoral vein in Vacutainer tubes. A standard canine questionnaire was applied and included questions about name, sex, age, size, color and main phenotypic features, birthplace, the age at which the pet entered the house, the dog's main function (hunt, company or protection) and the areas explored by the dog. The average domestic dog population was 4 years old. We considered dogs younger than one year as juveniles and older dogs as adults. Our sample included 95 dogs, composed of 68 adults and 27 juveniles ranging from 5 months to 16 years of age. Here, we considered each dog as representing a single event, even when related to the same house.

#### Triatomines

Triatomines were collected and identified by the International and National Laboratory of Reference for Triatominae Taxonomy, Oswaldo Cruz Institute, FIOCRUZ, Rio de Janeiro, Brazil. All collected triatomines were examined for the presence of flagellates by abdominal compression and observation of the insect feces by microscopy (fresh examination, 160× magnification). When trypomastigote forms were observed, the fecal material was inoculated into tubes containing Novy-MacNeal-Nicolle (NNN) medium with a Liver Infusion Tryptose (LIT) medium overlay. Before culture, and inside a biological security cabinet, the infected triatomines were immersed in the following solutions for three seconds: iodinated alcohol (0.003%), alcohol 70% and sterile saline (0.85%) supplemented with an antibiotic and antimycotic solution (10%).

### Parasitological and serological diagnostic procedures

The blood samples collected from small mammals and dogs were processed as follows: (i) a drop of blood was set between a glass slide and a coverslip for fresh blood examination; (ii) 0.6 ml of blood was cultured in two tubes containing NNN/LIT medium for hemoculture; and (iii) the remaining blood was centrifuged, and the serum or plasma obtained was stored at −20°C as material for serological assays. Fresh blood samples were immediately examined (160× magnification) for the presence of flagellates. The hemocultures were examined every other week for three (seronegative mammals) or five (seropositive mammals) months. Cultures from the triatomines were examined weekly for up to 120 days. Positive cultures were amplified for molecular characterization and cryopreservation and deposited in the “Trypanosoma from Sylvatic and Domestic Mammals and Vectors Collection”, Oswaldo Cruz Foundation – COLTRYP (Table S1).

Serological diagnoses were obtained using an adapted version of the indirect immunofluorescence antibody test (IFAT) described by Camargo [18]. The antigen used was an equal mixture of complete parasites derived from the strains I00/BR/00F (TcI) and MHOM/BR/1957/Y (TcII). Murinae rodents' sera were tested with anti-rat IgG; whereas dog's sera were tested with anti-dog IgG coupled to fluorescein isothiocyanate. Echimyidae rodents and marsupials were tested with the specific intermediary antibodies anti-*Thrichomys* sp. and anti-opossum, respectively, raised in rabbits, and the reaction was revealed by an anti-rabbit IgG conjugate. The cut-off value adopted was 1∶40 for domestic mammals and marsupials and 1∶10 for the rodents [19]. A confirmatory serological assay for the dogs' infection was conducted using an enzyme-linked immunosorbent assay (ELISA). The cut-off value for the ELISA was defined as the mean optical absorbance of the negative controls +20%, and to each reaction plate, we added 2 positive and 2 negative control sera. To evaluate possible cross-reactions and/or mixed infection by *T. cruzi* and *Leishmania* sp., dogs were also screened for *Leishmania* sp. infection using the rapid test for diagnosis of Canine Visceral Leishmaniasis (CVL) and an IFAT, the latter using antigens derived from a mixture of *Leishmania infantum* and *L. braziliensis* parasites. Dogs with serological titers higher for *Leishmania* sp. than for *T. cruzi* were considered as infected only by *Leishmania* sp. when *T. cruzi* titers were ≤1∶80 and as dually infected when titers were >1∶80, as defined elsewhere [20]. Animals were considered as infected by *T. cruzi* when serological analysis and/or hemoculture were positive. Dogs with negative hemocultures were considered infected by *T. cruzi* only when both the *T. cruzi* IFAT and ELISA were positive.

### Molecular characterization of the parasites

The positive cultures derived from mammals and triatomines were amplified in LIT medium at 28°C. Total genomic DNA was prepared from parasites in the logarithmic phase using standard phenol-chloroform protocols, as described elsewhere [21]. Multiplex PCR amplification of the mini-exon gene was performed under the conditions described by Fernandes [22]. For the identification of *T. cruzi* DTUs or mixed infections, bands of 150 bp (TcIII/TcIV), 200 bp (TcI) and 250 bp (TcII/TcV/TcVI), in addition to *T. rangeli* (TR: 100 bp), were considered [23]. A confirmatory characterization was also performed with PCR-RFLP 1f8/Alw21I [24]. Each reaction included negative and positive control samples from *T. cruzi* strains representing the six DTUs. PCR products were visualized in 2% agarose gel after ethidium bromide staining under ultraviolet light.

### Spatial and statistical analyses

The coordinates of the capture sites of mammals and vectors were determined using a hand-held Global Positioning System (GPS) receiver and recorded in the WGS 84 Datum (World Geodetic System 1984) geodetic coordinate system. The concordance between the IFAT-IgG and the ELISA was assessed by the kappa (k) statistic method, which measures agreement beyond chance. A κ value of more than 0.81 indicates excellent agreement; values from 0.61–0.8 and 0.41–0.6 indicate substantial and moderate agreement, respectively; and a value of less than 0.4 indicates poor agreement [25].

## Results

### Parasitological and serological surveys

#### *T. cruzi* infection in mammals and triatomines from the urban areas: Val-de-Cães and Jurunas

No small mammals were captured in any of the 20 mammal traps arranged in four residences in Jurunas. In Val-de-Cães, the small mammal fauna composition mainly comprised marsupial species (35/40 of the collected mammals). These mammals were exposed to *T. cruzi* infection (as indicated by positive serology), but their infection was sub-patent (no positive fresh blood examinations or hemocultures were obtained). *Philander opossum* was the most abundant mammalian species (57.5% of the collected mammals) and displayed a high prevalence of positive IFAT results (17/24, 70.8%). Six *Didelphis marsupialis* and five *Marmosa murina* were the other abundant mammalian species (each species representing 15% of the collected species) and both also displayed positive IFAT results (3 and 2 specimens, respectively, of each taxa). The distribution of the infected animals was not aggregated because animals exposed to *T. cruzi* were collected in all the established transects (Table 1).

**Table 1:** Capture and prevalence of infection by *Trypanosoma cruzi* of the small mammalian fauna examined in Val-de-Cães, and islands Combu, Murutucu and Pato, Belém municipality.

	Small mammalian fauna	Prevalence of infection
Localities	Order	Species	Capture (number of animals)	Positive IFAT	Positive Hemocultures	Parasite characterization
Val-de-Cães	Marsupialia	*Didelphis marsupialis*	6	3	0	
		*Philander opossum*	24	17	0	
		*Marmosa murina*	5	2	0	
	Rodentia	*Proechimys gr. goeldii*	1	0	0	
		*Rattus rattus*	3	1	0	
		*Dasyprocta prymnolopha*	1	n.d.	0	
	Pilosa	*Tamandua tetradactyla* *	1	n.d.	0	
Islands	Marsupialia	*Philander opossum*	7	7	6	TcI (4); *T. rangeli* (1); TcI/*T. rangeli* (1)
	Rodentia	*Proechimys gr. guianensis*	1	1	0	
		*Hylaeamys megacephalus*	1	0	0	
		**Total**	**50**	**31**	**6**	

**Footnotes:**

n.d = not determined;

*  = not included in the calculation of capture prevalence.

IFAT = Indirect Immunofluorescence Antibody Test.

Overall, *T. cruzi* infection in dogs was sub-patent both in the Val-de-Cães and in Jurunas (no positive hemocultures and/or infected fresh blood preparations were obtained). Dogs were exposed to *T. cruzi* infection in the two localities, as demonstrated by the presence of anti-*T. cruzi* antibodies. The prevalence of concomitant positive IFAT and ELISA results was 31.6% (6/19) in Val-de-Cães and 21.4% (3/14) in Jurunas (Table 2). The agreement between the IFAT-IgG and the ELISA was 83%, with a k value of 0.64 (substantial agreement).

**Table 2:** Prevalence of *Trypanosoma cruzi* infection by IFAT/ELISA and hemocultures in dogs examined in Val-de-Cães, Jurunas, and islands Combu, Murutucu and Pato, Belém municipality.

Localities	Seroprevalence (%)	Hemoculture prevalence (%)	Parasite characterization
Val-de-Cães	6/19 (31.6)	0/19	
Jurunas	3/14 (21.4)	0/14	
Combu	20/47 (42.5)	3/47 (6.4)	TcI (2); *T. rangeli* (1)
Murutucu	0/5	0/5	
Pato	5/9 (55.5)	0/9	
**Total**	**34/94 (36.2)**	**3/94 (3.2)**	

**Figure 2:**
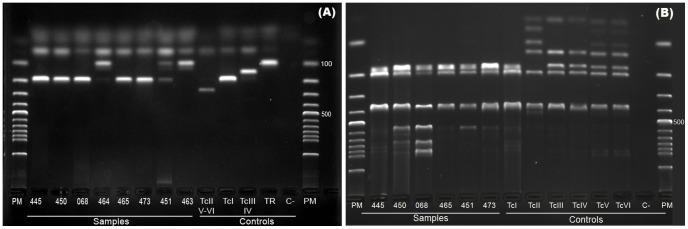
*Trypanosoma cruzi* genotyping of triatomine and mammalian isolates from the Belém/Pará, Brazil. (A) PCR products of the mini-exon gene analyzed by electrophoresis on na agarose gel stained with ethidium bromide. Lanes: M. molecular-weight markers (100 bp DNA ladder); control samples: TcI (200 bp), TcII/TcV/TcVI (250 bp), TcIII/TcIV (150 bp), and *T. rangeli* (100 bp). (B) PCR-RFLP products of 1f8 gene/Alw21I. Control samples: PCR-RFLP 1f8/Alw21I digestion patterns of TcI to TcVI: 445, 450 and 068: triatomine isolates (*Rhodinius pictipes* from Combu, Murutucu and Val-de-Cães, respectively) characterized as TcI; 464: dog isolate characterized as *T. rangeli*; 465: dog isolate characterized as TcI; 473: *Philander opossum* isolates characterized as TcI; 451: *P. opossum* isolate characterized as a mixed infection of TcI/*T. rangeli*; and 463: *P. opossum* isolate characterized as *T. rangeli*.

From the 17 triatomines collected in the urban area of the municipality, three were identified as *Panstrongylus lignarius*, and 14 were identified as *Rhodinius pictipes*, all of which were from Val-de-Cães. *O*nly three *R. pictipes* were positive for *T. cruzi* infection in fresh examination, two of which were characterized as TcI (Table 3, Figure 2).

**Table 3:** Prevalence of *Trypanosoma cruzi* infection in triatomines examined in Val-de-Cães, and islands Combu and Murutucu, Belém municipality.

Localities	Species (Triatomines)	Fresh examination/Total (%)	Positive Culture	Parasite characterization
**Val-de-Cães**	*Rhodnius pictipes*	3/14 (21.4)	2	TcI (2)
	*Panstrogylus lignarius*	0/3		
**Murutucu**	*R. pictipes*	6/35 (17.1)	5	TcI (7)
	*R. robustus*	2/8 (25)	2	
**Combu**	*R. pictipes*	30/239 (12.5)	30	TcI (29)
	*R. robustus*	7/62 (11.3)		
	Unidentified Nymph	14/35 (40)		
	**Total**	**62/396 (15.6%)**	**39**	**TcI (38)**

#### *T. cruzi* infection in mammals and triatomines from the Islands: Combu, Murutucu and Pato

Although still maintaining certain characteristics of the typical Amazonian forest, the islands display low richness and an abundance of wild mammalian species, indicating that this region has undergone a high degree of devastation. Overall, *Philander opossum* was the most abundant mammalian species (77.7%; N = 7/9), all of which were infected (positive IFAT). Additionally, *T. cruzi* isolates were obtained from 71.4% (5/7) of this species showing their high reservoir competence. In addition to *P. opossum*, only one *Proechimys gr*. *guianensis* and one *Hylaeamys megacephalus* were captured on Combu and Pato Islands, respectively. However, despite the positive serology observed in the *P. gr. guianensis*, no positive hemocultures were obtained from this rodent. All of the *T. cruzi* isolates were characterized as TcI, including one mixed TcI/*T. rangeli* infection. One isolate obtained from another *P. opossum* was characterized as only *T. rangeli* (Table 1, Figure 2).

Dogs were exposed to *T. cruzi* infection on Combu and Pato Islands, displaying serological rates that ranged from 42.5% (N = 20/47) to 55.5% (N = 5/9), respectively (Table 2). Two *T. cruzi* isolates were obtained from dogs from Combu Island, both characterized as TcI. Another dog from the same Island that displayed a positive hemoculture was infected by *T. rangeli* (Table 2, Figure 2). Three of the five serologically positive dogs from Pato Island were juveniles (less than 1 year). Only five dogs were examined on Murutucu Island none of which were infected by *T. cruzi*.

We collected a total of 379 triatomines, and 59 from Combu (51/336) and Murutucu (8/43) Islands were positive in the fresh feces examination. The triatomines were identified as *Rhodinius pictipes* (N = 274) and *R. robustus* (N = 70), in addition to 35 unidentified nymphs. All of the characterized isolates were defined as TcI (Table 3).

## Discussion

*Trypanosoma cruzi*, rarely reported to be transmitted to humans by the oral route in the past, is currently responsible for frequent outbreaks of acute cases of Chagas disease contracted orally and characterized by high morbidity and mortality. *T. cruzi* is currently considered to be the most prominent food-borne parasite among the emerging protozoan parasites [26]. In this new epidemiological scenario, the most common approach of the health authorities has been to attempt to demonstrate the “source” of the “new” human diseases, rather than determining “why” in terms of One Health. In such cases, we need to better understand the factors that enable zoonotic transmission from wildlife to humans, and which factors may lead to outbreaks of disease [27].

Examining host-parasite interactions from the perspective of complex and interdependent systems is the most appropriate way to understand the epidemiology of transmission cycles. This understanding is particularly important in the case of *T. cruzi*, a multi-host parasite immersed in intricate transmission cycles and capable of infecting its hosts by different routes. One of the major reasons for needing to understand the complex transmission of *T. cruzi*, if prevention of Chagas disease is the objective, is the identification of the mammalian species that are acting as reservoirs in the transmission areas. This topic should be studied in a broad and multidisciplinary manner that involves the knowledge of the mammalian diversity of the area (potential reservoirs of the parasite), the peculiarities of the host-parasite interaction (including the ecological characteristics of each one) and the factors that may favor contact between infected mammals and vectors, which ultimately modulate the dynamics of *T. cruzi* transmission. Evaluation of the local characteristics of enzootic *T. cruzi* transmission in two areas and the habits of the local population, including their mode of interaction with the environment, led us to propose a new mechanism of *T. cruzi* dispersion, which we call *Distantiae* transmission.

Humans were likely included in the *T. cruzi* transmission cycle as soon as they arrived in the Americas. Several epidemiological variations have likely occurred during this longstanding host-parasite interaction. In Brazil, three important landmarks concerning Chagas disease can be considered: (i) Chagas infection and disease in prehistory, as proven by the discovery of *T. cruzi* DNA in a mummy with megacolon that was dated as approximately 560±40 years ago, and in a bone piece dated between 4,500 and 7,000 years ago, showing that Chagas disease in Brazil most likely preceded European colonization and occurred in humans largely before *T. infestans* domiciliation; (ii) *T. cruzi* transmission to humans attributed to the domiciliation of an exotic triatomine species (*T. infestans*) that adapted to the precarious mud dwellings built after European colonization [28]; and (iii) the current epidemiological scenario, especially in the Brazilian Amazon region, where domiciled triatomines have not been reported and the numbers of human cases have been increasing due to oral infections [29], [30]. Amazonia represents a mosaic of landscapes, cultures and ethnicities. Thus, even in the previously non-endemic Chagas disease area of Amazonia, distinct epidemiological scenarios of *T. cruzi* transmission have been described. Examples include transmission by *Rhodnius brethesi* to humans during the piaçava harvest, familiar contamination during palmetto extraction and açaí and bacaba juice contamination in areas that face robust enzootic *T. cruzi* transmission between vectors and wild mammals and vectors [9], [11], [29], [31]–[33]. Each of these scenarios is related to different cultural habits, routes of infection, infective forms and mechanisms of parasite transmission, and each demands different solutions. The situation observed in Belém does not fit in any of these scenarios described above, but in fact, this municipality displayed the highest number of recurrent cases of ACD between 2006 and 2011. Here, in addition to the various epidemiological situations observed in the Amazon region, we demonstrate an additional situation, in which humans from one area were infected by contact with infected vectors coming from another area. This new scenario also requires a different look at the surveyed areas.

Aiming to understand the origin of the ACD cases in Belém, we first investigated the enzootic transmission cycle of *T. cruzi* in the two suburbs that presented the majority of ACD cases. In Val-de-Cães, we observed the presence of *T. cruzi* transmission in the residual forest that can still be found in the area. However, only by low *T. cruzi* seroprevalence was observed in small mammals and dogs, and few triatomines were infected with *T. cruzi*. This is not the common situation observed in other areas of ACD transmission in Pará state, which are always associated with a high prevalence of positive hemocultures among wild mammals and vectors [9], [20], [33]. Despite the fact that the scenario does not explain the great number of ACD cases, the unlikely hypothesis of autochthonous transmission cannot be excluded. However, if the hypothesis is unlikely in Val-de-Cães, it is impossible to think that cases of ACD in Jurunas are autochthonous. Our data clearly show that cases of ACD in Jurunas originate from an external source. There is no important enzootic cycle of *T. cruzi* in the area, as signaled by the absence of small mammal capture due to the high degree of devastation in the forest fragments. Among the seropositive dogs from Jurunas, two cases of seropositivity may have been due to a cross-reaction (titer 1/40), and the only dog with a titer of 1/160 may have been infected in another location because that dog was 5 years old and the owner was not informed about the dog's movement or the places where the dog lived before. This scenario, in which there were no signs of infection with *T. cruzi* in the animals in the neighborhood of Jurunas, indicates that a different source/reservoir of *T cruzi* must exist.

To prove *Distantiae* transmission, we analyzed the cultural aspects (movements of people and materials) of the local population that could be associated with the ACD cases observed in Jurunas and Val-de-Cães. We already knew that the ACD cases in the urban portion of Belém were always associated with the oral consumption of açaí juice [34]. Notably, Jurunas is a neighborhood located on the banks of the Guamá river, which is the main route of access to the metropolitan region for the boats that transport and supply the açaí fruit that are harvested on the islands (Combu and Murutucu), which are approximately 20 minutes apart by boat, each day. We decided to analyze the enzootic transmission cycle of *T. cruzi* in the places where these fruits are harvested and the logistics of their transportation and distribution.

The enzootic scenario observed on the islands strongly supports our hypothesis that the origin of the ACD cases in the metropolitan area of Belém is distant from that area. The islands are characterized by a low abundance of wild mammals, indicating a high degree of environmental devastation. Moreover, *T. cruzi* transmission occurs close to houses, as signaled by *T. cruzi* isolation from dogs and small mammals captured in peridomiciliary areas. The presence of infected young mammals (dogs and small wild mammals) indicates that on the islands infection is recent, and transmission is active. All of the triatomines collected on the Islands came from areas of açaí harvesting, which are surrounded by the areas where infected dogs, rodents and marsupials were examined, suggesting that these regions present an expressive *T. cruzi* transmission cycle, with a high number of infected bugs.

*T. cruzi* isolates from vectors and wild and domestic mammals were all characterized as TcI, which is the DTU associated with human infection in this area. Dogs have already been suggested to provide a link between the domestic and the wildlife cycles of transmission [35] and have also been demonstrated to be very useful as sentinels of *T. cruzi* transmission areas, inclusive of the Amazon region [20], [36].

Our findings indicate that the ACD cases in the metropolitan region of Belém are related to the infected sylvatic triatomine bugs accidentally transported from the islands. These insects are derived from the islands where we observed a robust enzootic *T. cruzi* transmission cycle, and the parasite is transmitted to humans due to the inadequate preparation of açaí juice. The large volume of baskets and the mobility of them among several sale points demonstrated to be a limiting condition that enabled us to investigate every baskets that arrived to Belém during our study. Because of that and mainly because the epidemiological investigations of Belém cases already pointed towards the origin of fruit, the search of triatomines in fruit baskets was not included among our objectives. Moreover, the transport of insects in fruit baskets was already demonstrated in the Amazonian Abaetetuba municipality by Valente and coauthors [16]. They searched for triatomines on boats docked near islands and collected 12 triatomines, 5 of which had flagellates in their feces. One dead bug with viable parasites was also found in a fruit basket coming from the island [16]. Later, Valente and coauthors [37] also described a simulation in which non-infected triatomines from their colony were added to a basket of açaí, and the basket's route was followed to açaí juice preparation. This simulation was repeated 15 times, and only once did the professionals identify the insect. It is noteworthy that this transmission can occur with the consumption of not only freshly made juice, but also frozen juice [34], [38]. These authors demonstrated that infective *T. cruzi* can survive in açaí pulp at room temperature for 48 h, at 4°C for 144 h and at −20°C for 26 h.

In the Amazon region, food contamination by infected house-invading triatomines is the result of cultural characteristics, a high prevalence of infected bugs and the unhygienic manipulation of foodstuffs. Obviously, most cases of ACD in Belém should be prevented by the control of the baskets that carry the fruits to the metropolitan region of Belém and by the implementation of health education programs. These programs should include information about standardized basic hygiene procedures in food handling, and especially açaí handling to prevent contamination during preparation of the pulp. However, this approach is far from simple because Belém has approximately 3500 sites of açaí juice sales, most of which are temporary, *i.e.*, they are not fixed and display a seasonal distribution each year. The professionals that prepare the açaí juice are also temporary and despite the periodical sanitary inspection of these settlements, every year, approximately half of them are replaced by individuals who are working for the first time. Even considering these logistics, which make the control of *T. cruzi* transmission to humans very difficult, there is no better method to prevent new cases.

Based on our results, we conclude that the autochthonous cases of Chagas in Belém city were due to the intake of açaí juice prepared in low hygienic conditions, from fruits harvested and transported in baskets together with *T. cruzi* infected triatomines from nearby islands. Moreover, this peculiar and new epidemiological pattern emphasizes the importance of unraveling all components of this complex, multifactorial, unpredictable and dynamic system that underlie the emergence of the human cases in the different localities of its occurrence. This means that generalizing control measures will probably result in failure.

## Supporting Information

Table S1 Data of Host species captured in each area, code from the *Trypanosoma cruzi* isolates from Belém/Pará State deposited in the “Trypanosoma from Sylvatic and Domestic Mammals and Vectors Collection”, Oswaldo Cruz Foundation (COLTRYP), geographic origin and lineage.
(DOC)

## References

[1] Chagas C (1909) Nova tripanozomiaze humana. Estudos sobre a morfolojia e o ciclo evolutivo do Schizotrypanum cruzi n. gen., n. sp., ajente etiolojico de nova entidade morbida do homem. Mem Inst Oswaldo Cruz 1: 159–218.

[2] Moreira D, López-García P, Vickerman K (2004) An updated view of kinetoplastid phylogeny using environmental sequences and a closer outgroup: proposal for a new classification of the class Kinetoplastea. Int J Syst Evol Micr 54: 1861–1875 Doi: 10.1099/ijs.0.63081-0. 10.1099/ijs.0.63081-0 15388756

[3] Carrasco HJ, Segovia M, Llewellyn MS, Morocoima A, Urdaneta-Morales S, et al (2012) Geographical Distribution of *Trypanosoma cruzi* Genotypes in Venezuela. PLoS Negl Trop Dis 6 (6) e1707 10.1371/journal.pntd.000170 22745843 PMC3383755

[4] Jansen AM, Roque ALR (2010) Domestic and wild mammalian reservoir. In: Telleria J, Tibayrenc M, editors. American trypanosomiasis Chagas Disease - one hundred years of research. London: Elsevier. 249–276.

[5] Zingales B, Andrade SG, Briones MR, Campbell DA, Chiari E, et al (2009) A new consensus for *Trypanosoma cruzi* intraspecific nomenclature: second revision meeting recommends TcI to TcVI. Mem Inst Oswaldo Cruz 104: 1051–1054. 20027478 10.1590/s0074-02762009000700021

[6] Zingales B, Miles MA, Campbell DA, Tibayrenc M, Macedo AM, et al (2012) The revised *Trypanosoma cruzi* subspecific nomenclature: rationale, epidemiological relevance and research applications. Infect Genet Evol 12: 240–253. 22226704 10.1016/j.meegid.2011.12.009

[7] Marcili A, Lima L, Cavazzana M, Junqueira AC, Veludo HH, et al (2009) A new genotype of *Trypanosoma cruzi* associated with bats evidenced by phylogenetic analyses using SSU rDNA, cytochrome b and Histone H2B genes and genotyping based on ITS1 rDNA. Parasitology 136: 641–655. 19368741 10.1017/S0031182009005861

[8] Ramirez JD, Duque MC, Montilla M, Cucunuba ZM, Guhl F (2012) Multilocus PCR-RFLP profiling in *Trypanosoma cruzi* I highlights an intraspecific genetic variation pattern. Infect Genet Evol 12: 1743–1750. 22824418 10.1016/j.meegid.2012.06.018

[9] Roque ALR, Xavier SCC, Gerhardt M, Silva MFO, Lima VS, et al (2013) *Trypanosoma cruzi* among wild and domestic mammals in different areas of the Abaetetuba municipality (Pará State, Brazil), an endemic Chagas disease transmission area. Vet Parasitol 193: 71–77. 23261089 10.1016/j.vetpar.2012.11.028

[10] Aguilar HM, Abad-Franch F, Dias JCP, Junqueira ACV, Coura JR (2007) Chagas disease in the Amazon Region. Mem Inst Oswaldo Cruz 102 (Suppl. I) 47–55. 17891274 10.1590/s0074-02762007005000098

[11] Coura JR, Junqueira ACV (2012) Risks of endemicity, morbidity and perspectives regarding the control of Chagas disease in the Amazon Region. Risks of endemicity, morbidity and perspectives regarding the control of Chagas disease in the Amazon Region. Mem Inst Oswaldo Cruz 107 (2) 145–154. 22415251 10.1590/s0074-02762012000200001

[12] Shaw J, Lainson R, Fraiha H (1969) Considerações sobre a epidemiologia dos primeiros casos autóctones de doença de Chagas registrados em Belém, Pará, Brasil. Rev Saúde Pública 3: 153–157. 4984569

[13] Abad-Franch F, Diotaiuti L, Gurgel-Gonçalves R, Gürtler RE (2013) Certifying the interruption of Chagas disease transmission by native vectors: cui bono? Mem Inst Oswaldo Cruz 108 (2) 251–254. 23579810 10.1590/0074-0276108022013022 PMC3970656

[14] Valente SAS, Valente VC, César MJB, Santos MP (1997) Registro de 15 casos autóctones de doença de Chagas no Estado do Amapá com evidências de transmissão oral. Congresso da Sociedade Brasileira de Medicina Tropical, XXXIII, TL 056, 53

[15] Valente VC, Pinto AYN, Valente SAS (2000) Novo episódio familiar com 7 casos de doença de Chagas aguda e autóctone em Bagre Estado do Pará. Congresso da Sociedade Brasileira de Medicina Tropical, XXXVI, TL 113, Rev Soc Bras Med Trop 33 (Supl.I) 388–389.

[16] Valente SAS, Valente VC, Pinto AYN (2002) Por que ocorrem episódios familiares de doença de Chagas associado à transmissão oral na Amazônia brasileira? Revista da Sociedade Brasileira de Medicina Tropical 35 (I) 165 In: Anais do 38^a^ Congresso da Sociedade Brasileira de Medicina Tropical.

[17] Bonvicino CR, Otazu IB, D'Andrea PS (2002) Karyologic evidence of diversification of the genus *Thrichomys* (Rodentia, Echimyidae). Cyt Gen Res 97: 200–204. 10.1159/000066613 12438714

[18] Camargo ME (1966) Fluorescent antibody test for the serodiagnoses of American Trypanosomiasis: technical modification employing preserved culture forms of *Trypanosoma cruzi* in a slide test. Rev Inst Med Trop São Paulo 8: 227–234. 4967348

[19] Herrera L, D'Andrea PS, Xavier SCC, Mangia RH, Fernandes O, et al (2005) *Trypanosoma cruzi* infection in wild mammals of the National Park “Serra da Capivara”, and its surroundings (Piauí, Brazil), endemic for Chagas disease. Trans R Soc Trop Med Hyg 99: 379–388. 15780345 10.1016/j.trstmh.2004.07.006

[20] Xavier SCC, Roque ALR, Lima VS, Monteiro KJL, Otaviano JCR, et al (2012) Lower Richness of Small Wild Mammal Species and Chagas Disease Risk. PLoS Negl Trop Dis 6 (5) e1647 10.1371/journal.pntd.0001647 22616021 PMC3352825

[21] Vallejo GA, Guhl F, Chiari E, Macedo AM (1999) Species specific detection of *Trypanosoma cruzi* and *Trypanosoma rangeli* in vector and mammalian hosts by polymerase chain reaction amplification of kinetoplast minicircle DNA. Acta Trop 72: 203–212. 10206119 10.1016/s0001-706x(98)00085-0

[22] Fernandes O, Santos SS, Cupolillo E, Mendonca B, Derre R, et al (2001) A mini-exon multiplex polymerase chain reaction to distinguish the major groups of *Trypanosoma cruzi* and *T. rangeli* in the Brazilian Amazon. Trans R Soc Trop Med Hyg 95: 97–99. 11280078 10.1016/s0035-9203(01)90350-5

[23] Aliaga C, Breniere SF, Barnabe C (2011) Further interest of miniexon multiplex PCR for a rapid typing of *Trypanosoma cruzi* DTU groups. Infect Genet Evol 11: 1155–1158. 21255686 10.1016/j.meegid.2010.11.013

[24] Rozas M, De DS, Adaui V, Coronado X, Barnabe C, et al (2007) Multilocus polymerase chain reaction restriction fragment–length polymorphism genotyping of *Trypanosoma cruzi* (Chagas disease): taxonomic and clinical applications. J Infect Dis 195: 1381–1388 10.1086/513440 17397011

[25] Viera AJ, Garrett JM (2005) Understanding Interobserver Agreement: The Kappa Statistic. Fam Med 37 (5) 360–3. 15883903

[26] Yoshida N, Tyler KM, Llewellyn MS (2011) Invasion mechanisms among emerging food-borne protozoan parasites. Trends in Parasitol 27 (10) 459–466. 10.1016/j.pt.2011.06.006 21840261

[27] Thompson RC (2013) Parasite zoonoses and wildlife: One Health, spillover and human activity. Int J Parasitol 43 (12–13) 1079–88 10.1016/j.ijpara.2013.06.007 23892130 PMC7126848

[28] Fernandes A, Iñiguez AM, Lima VS, Souza SMFM, Ferreira LF, et al (2008) Pre-Columbian Chagas disease in Brazil: *Trypanosoma cruzi* I in the archaeological remains of a human in Peruaçu Valley, Minas Gerais, Brazil. Mem Inst Oswaldo Cruz 103 (5) 514–516. 18797771 10.1590/s0074-02762008000500021

[29] Pinto AY, Valente SA, Valente VC, Ferreira JAG, Coura JR (2008) Acute phase of Chagas disease in the Brazilian Amazon region: study of 233 cases from Pará, Amapá and Maranhão observed between 1988 and 2005. Rev Soc Bras Med Trop 41 (6) 602–614. 19142440 10.1590/s0037-86822008000600011

[30] Shikanai-Yasuda MA, Carvalho NB (2012) Oral Transmission of Chagas Disease. Emerg Infect 54: e845. 10.1093/cid/cir956 22238161

[31] Beltrão Hde B, Cerroni Mde P, Freitas DR, Pinto AY, Valente Vda C, et al (2009) Investigation of two outbreaks of suspected oral transmission of acute Chagas disease in the Amazon region, Pará State, Brazil, in 2007. Trop Doct 39 (4) 231–2 10.1258/td.2009.090035 19762577

[32] Nóbrega AA, Garcia MH, Tatto E, et al (2009) Oral transmission of Chagas disease by consumption of açaí palm fruit, Brazil. Emerg Infect Dis 15: 653–655. 19331764 10.3201/eid1504.081450 PMC2671433

[33] Roque AL, Xavier SCC, Rocha MG, Duarte AC, D'Andrea PS, et al (2008) *Trypanosoma cruzi* transmission cycle among wild and domestic mammals in three areas of orally transmitted Chagas disease outbreaks. Am J Trop Med Hyg 79: 742–749. 18981516

[34] Passos LAC, Guaraldo AMA, Barbosa RL, Dias VL, Pereira KS, Set al (2012) Survival and infectivity of *Trypanosoma cruzi* in açaí pulp: in vitro and in vivo study. Epidemiol Serv Saúde 21 (2) 223–232 10.5123/S1679-49742012000200005

[35] Ramirez JD, Turriago B, Taipa-Calle G, Guhl F (2013) Understanding the role of dogs (*Canis lupus familiaris*) in the transmission dynamics of *Trypanosoma cruzi* genotypes in Colombia. Vet Parasitol 196 (1–2) 216–9 10.1016/j.vetpar.2012.12.054 23351975

[36] Roque ALR, Jansen AM (2008) The importance of sentinel domestic animals to identify risk areas to the emergence of Chagas disease. Rev Soc Bras Med Trop 41 (Supl III) 191–193.

[37] Valente SA (2009) Centenário da Descoberta da Doença de Chagas (1909–2009): Voo e contaminação. Simpósio Internacional Radis N° 81

[38] Barbosa RL, Dias VL, Pereira KS, Schmidt FL, Franco RM, et al (2012) Survival in vitro and virulence of *Trypanosoma cruzi* in açaí pulp in experimental acute Chagas disease. J Food Prot 75 (3) 601–6 10.4315/0362-028X.JFP-11-233 22410239

