# Supplementary material for: Distantiae Transmission of Trypanosoma cruzi: A New Epidemiological Feature of Acute Chagas Disease in Brazil
Source: PLoS Negl Trop Dis. 2014 May 22;8(5):e2878. doi: 10.1371/journal.pntd.0002878 (PMC4031066; doi:10.1371/journal.pntd.0002878)
Supplement: Table S1 — Data of Host species captured in each area, code from the Trypanosoma cruzi isolates from Belém/Pará State deposited in the “Trypanosoma from Sylvatic and Domestic Mammals and Vectors Collection”, Oswaldo Cruz Foundation (COLTRYP), geographic origin and lineage. (DOC) [file pntd.0002878.s001.doc]

| **COLTRYP code *** | **Host** | **Geographic origin** | **Lineage**** |
| --- | --- | --- | --- |
| 068 | *Rhodnius pictipes* | Val-de-Cães | TcI |
| 065 | *R. pictipes* | Val-de-Cães | TcI |
| 449 | *Philander opossum* | Murutucu | TcI |
| 451 | *P. opossum* | Murutucu | TcI /TR |
| 427 | *Rhodnius pictipes* | Murutucu | TcI |
| 430 | *R. pictipes* | Murutucu | TcI |
| 432 | *R. pictipes* | Murutucu | TcI |
| 443 | *R. pictipes* | Murutucu | TcI |
| 452 | *R. pictipes* | Murutucu | TcI |
| 450 | *Rhodnius robustus* | Murutucu | TcI |
| 429 | *R. robustus* | Murutucu | TcI |
| 465 | *Canis familiaris* | Combu | TcI |
| 470 | *C. familiaris* | Combu | TcI |
| 464 | *C. familiaris* | Combu | TR |
| 461 | *Philander opossum* | Combu | TcI |
| 462 | *P. opossum* | Combu | TcI |
| 473 | *P. opossum* | Combu | TcI |
| 463 | *P. opossum* | Combu | TR |
| 423 | *Rhodnius* sp. | Combu | TcI |
| 424 | *Rhodnius* sp. | Combu | TcI |
| 428 | *Rhodnius* sp. | Combu | TcI |
| 431 | *Rhodnius* sp. | Combu | TcI |
| 434 | *Rhodnius* sp. | Combu | TcI |
| 435 | *Rhodnius* sp. | Combu | TcI |
| 436 | *Rhodnius* sp. | Combu | TcI |
| 438 | *Rhodnius* sp. | Combu | TcI |
| 441 | *Rhodnius* sp. | Combu | TcI |
| 444 | *Rhodnius* sp. | Combu | TcI |
| 445 | *Rhodnius* sp. | Combu | TcI |
| 425 | *Rhodnius pictipes* | Combu | TcI |
| 426 | *R. pictipes* | Combu | TcI |
| 437 | *R. pictipes* | Combu | TcI |
| 439 | *R. pictipes* | Combu | TcI |
| 440 | *R. pictipes* | Combu | TcI |
| 442 | *R. pictipes* | Combu | TcI |
| 447 | *R. pictipes* | Combu | TcI |
| 448 | *R. pictipes* | Combu | TcI |
| 454 | *R. pictipes* | Combu | TcI |
| 455 | *R. pictipes* | Combu | TcI |
| 456 | *R. pictipes* | Combu | TcI |
| 457 | *R. pictipes* | Combu | TcI |
| 458 | *R. pictipes* | Combu | TcI |
| 459 | *R. pictipes* | Combu | TcI |
| 460 | *R. pictipes* | Combu | TcI |
| 433 | *Rhodnius robustus* | Combu | TcI |
| 446 | *R. robustus* | Combu | TcI |
| 453 | *R. robustus* | Combu | TcI |

**Supporting Information**

**Table S1.** ***Trypanosoma cruzi*** **genotyping of isolates from the Belém municipality/Pará State, Brazil.** Mini-exon multiplex PCR products, 1f8 gene/Alw21I PCR-RFLP products.

**Legend S1:**

Data of host species captured in each area, code from the *Trypanosoma cruzi* isolates from Belém/Pará State deposited in the “Trypanosoma from Sylvatic and Domestic Mammals and Vectors Collection”, Oswaldo Cruz Foundation (COLTRYP), geographic origin and lineage.

**Footnotes:**

TcI: *Trypanosoma cruzi* DTU I; TR: *Trypanosoma rangeli*

* Code number of the isolates in COLTRYP

** Genotyped using the method developed by Fernandes [22], Rozas [24]
